# Therapist perceptions of the implementation of a new screening procedure using the ItFits-toolkit in an iCBT routine care clinic: A mixed-methods study using the consolidated framework for implementation research

**DOI:** 10.3389/fpsyt.2023.1104301

**Published:** 2023-04-06

**Authors:** Kristine Tarp, Søren Lange Nielsen, Trine Theresa Holmberg, Caroline Høier Dalsgaard, Simone Borkner, Helene Skaarnes, Esben Kjems Jensen, Jordi Piera-Jiménez, Christiaan Vis, Kim Mathiasen

**Affiliations:** ^1^Research Unit for Digital Psychiatry, Department of Clinical Research, University of Southern Denmark, Odense, Denmark; ^2^Centre for Digital Psychiatry, Mental Health Services in the Region of Southern Denmark, Odense, Denmark; ^3^Catalan Health Service, Barcelona, Spain; ^4^Digitalization for the Sustainability of the Healthcare System DS3-IDIBELL, L'Hospitalet de Llobregat, Barcelona, Spain; ^5^Faculty of Informatics, Multimedia and Telecommunications, Universitat Oberta de Catalunya, Barcelona, Spain; ^6^Clinical, Neuro- & Developmental Psychology, Faculty of Behavioural and Movement Sciences, VU Amsterdam, Amsterdam, Netherlands; ^7^Amsterdam Public Health Research Institute, Amsterdam, Netherlands; ^8^Section for Research-Based Innovation, Division of Psychiatry, Haukeland University Hospital, Bergen, Norway

**Keywords:** therapist perceptions, implementation, anxiety, depression, iCBT, NPT, CFIR

## Abstract

**Introduction:**

This study investigates the implementation of a new, more automated screening procedure using the ItFits-toolkit in the online clinic, Internet Psychiatry (iPsych) (www.internetpsykiatrien.dk), delivering guided iCBT for mild to moderate anxiety and depressive disorders. The study focuses on how the therapists experienced the process.

**Methods:**

Qualitative data were collected from semi-structured individual interviews with seven therapists from iPsych. The interviews were conducted using an interview guide with questions based on the Consolidated Framework for Implementation Research (CFIR). Quantitative data on the perceived level of normalization were collected from iPsych therapists, administrative staff, and off-site professionals in contact with the target demographic at 10-time points throughout the implementation.

**Results:**

The therapists experienced an improvement in the intake procedure. They reported having more relevant information about the patients to be used during the assessment and the treatment; they liked the new design better; there was a better alignment of expectations between patients and therapists; the patient group was generally a better fit for treatment after implementation; and more of the assessed patients were included in the program. The quantitative data support the interview data and describe a process of normalization that increases over time.

**Discussion:**

The ItFits-toolkit appears to have been an effective mediator of the implementation process. The therapists were aided in the process of change, resulting in an enhanced ability to target the patients who can benefit from the treatment program, less expenditure of time on the wrong population, and more satisfied therapists.

## 1. Introduction

The prevalence and burden of anxiety and depressive disorders are high (1–4) and increasing because of the growth and aging of the population (5). According to the 2017 Global Burden of Diseases, Injuries, and Risk Factors Study (GBD) (6), the burden of mental disorders is present in both sexes and across all age groups. Mental disorders have consistently accounted for more than 14% of age-standardized years lived with disability (YLD) for nearly three decades. The same study has found anxiety disorders to be a top cause of non-fatal burden for women, and depressive disorders were one of the three leading causes of non-fatal health loss for nearly three decades.

One reason why this burden persists is due to the treatment gap between the number of people with mental disorders and the number of people being treated for mental disorders (7, 8). Even though effective treatment exists (9), the reach of face-to-face cognitive behavioral therapy (CBT) is limited (10), and only about half of the people suffering from mental disorders in Europe receive the needed professional help (11). This may be due to general practitioners' (GPs) low knowledge about and referral rates to CBT (12). GPs are often the first contact in the pathway to care and are therefore considered crucial in the management of mental disease (13). Additionally, 70% of people with mental health problems do not consult their GP about their issues and therefore do not have access to CBT (14). A reason why people do not seek their GPs can be explained by the fact that many people view mental health problems as something they have to manage themselves (12).

In a recent dynamic modeling analysis, Skinner et al. (15) found that mental health in the population improves with increased access to treatment. This may point to the fact that addressing the substantial and persistent treatment gap should remain a global public health priority. To help reduce this gap, Internet-based CBT (iCBT) may be a solution. Several studies have demonstrated the effectiveness of iCBT as a treatment for mild to moderate anxiety and depressive disorders (16–21). Findings from an observational study evaluating the clinical effectiveness of iCBT for mild to moderate anxiety and depressive disorders in routine secondary care support the hypothesis that iCBT can help bridge the gap between the need for treatment and its provision (22). In addition, an implementation study process evaluating iCBT within community mental health clinics identified iCBT as a more convenient method of receiving care, where therapists had positive perceptions of the treatment mode and strongly agreed that citizens should have access to it (23).

To bridge this treatment gap between people with mental disorders and the number being treated, the successful implementation of iCBT is crucial, as poor implementation may contribute to the currently limited uptake numbers (24). The normalization process theory (NPT) defines implementation as an intentional, strategic, and planned process where normalization can occur as innovation becomes integrated/embedded in an organization (25). During such an implementation of iCBT, guiding tools may be helpful remedies for a successful process, as they may assist in identifying and realizing projects to further enhance implementation. Therefore, we found it relevant to use a generic, Integrated Theory-based Framework for Intervention Tailoring Strategies for data-driven, tailored implementation of evidence-based eHealth services (ItFits-toolkit). ItFits-toolkit is an NPT-informed process provided on an online platform that aims to support implementers in developing, applying, and monitoring implementation strategies adapted to the local context. The ItFits-toolkit may have the potential to influence implementation at different levels (24).

According to a systematic review of barriers and facilitating factors to the implementation of eMental Health for mood disorders in routine practice (26), empirical evidence on the effects of implementation strategies that address barriers and facilitators is warranted to contribute to the understanding of the mechanisms in implementation processes. In this regard, the Consolidated Framework for Implementation Research (CFIR) is a helpful framework for elucidating the barriers and facilitators that influence implementation (27) and for identifying recommendations for improving the uptake and implementation of iCBT in clinical settings (23). Integrating CFIR into both the data collection and analysis phases and in the selection of relevant constructs for deployment can be especially helpful in advancing implementation science (28).

The implementation of an optimized screening procedure can be seen as a key component for iPsych to be able to reach the target population, namely patients with mild to moderate anxiety or depression. Screening for the right demographic is especially important in a clinical setting. Having a scope of inclusion that is too broad for the treatment paradigm, means including people for whom the treatment may not be appropriate. This is a waste of resources and can lead to dissatisfaction for patients, therapists, and the site itself. On the other hand, a scope that is too narrow means excluding people who may benefit from the treatment. Therefore, optimizing intake can ensure the quality of the treatment. However, any change to the status quo requires resources to retrain therapists and support staff in daily routines. Consequently, using dedicated time and energy to improve the implementation process of the needs and specifications of the screening program could potentially reduce resource expenditure and increase satisfaction with the program, while disrupting the status quo as little as possible. Therefore, the present study aims to investigate perceptions of the implementation of a new more automated screening procedure using the ItFits-toolkit in a specialized clinic delivering guided iCBT for mild to moderate anxiety and depressive disorders.

## 2. Materials and methods

### 2.1. Design

The present study employed a mixed-methods design, utilizing an embedded explanatory sequential approach, in order to examine the therapists' perceptions of the implementation of the new screening procedure. Specifically, the study employed a combination of semi-structured interviews with therapists and surveys sent to therapists and other professionals with a referring role.

The embedded design is a mixed-methods approach that uses one data set in a supporting, secondary role in a study that is primarily based on other types of data. The underlying principle of this design is that a single data set is not sufficient to answer all research questions and that different types of questions require different types of data. The premises of this design are that a single data set is inadequate, that multiple research questions need to be addressed, and that each question necessitates different types of data (29). In this study, the quantitative data were used as secondary and complementary data to the primary qualitative data because the quantitative data set was deemed too small to sufficiently answer the research question on its own. This approach allowed for a more comprehensive understanding and evaluation of the implementation and normalization of the intervention, by utilizing both quantitative and qualitative data, and it provided a more nuanced understanding of the research question (29).

### 2.2. Setting

The Internet Psychiatry Clinic (iPsych) is operated by the Center for Digital Psychiatry (CEDIP) at the Mental Health Services in the Region of Southern Denmark. In 2013, iPsych started on a trial basis in the Region of Southern Denmark and has subsequently been operated on a temporary basis from 2015 until it became a permanent service in the region in 2019. Between 2018 and 2020, iPsych was rolled out nationally on a trial basis up until it became a permanent national offer in 2021.

iPsych is part of routine care in Denmark. Treatment is publicly funded and free for the patients. Patients self-refer for treatment *via* a website through a secure application platform. Self-referral has been found to enable people with mental health disorders to access mental health services without the need for a referral from their GP (14). Patients authenticate their identity by logging in with their unique civil registration number. The applications are screened, first through a request form and then by trained psychologists. The inclusion criteria are as follows: ≥18 years of age and meeting the diagnostic criteria for mild to moderate depressive disorder, single phobia, social phobia, or panic disorder with or without agoraphobia, based on the criteria from the International Classification of Diseases, 10th revision (30). Exclusion criteria are as follows: diagnosed personality disorders; severe anxiety or depression; ongoing psychological treatment elsewhere; schizophrenia, bipolar disorder, or related disorders; post-traumatic stress disorder (PTSD); obsessive-compulsive disorder (OCD) as a primary disorder; alcohol or drug abuse; or enhanced risk of suicide.

Unless applicants clearly indicate on the screening questionnaire that they meet one or more exclusion criteria, they will be invited to a video-based assessment interview. The video assessment uses the Mini-International Neuropsychiatric Interview (MINI) (31) to assess eligibility and is conducted by either a properly licensed or supervised psychologist. Following the video-based assessment, eligible patients are invited to participate in an iCBT program. The iCBT program consists of sessions that offer psychoeducation and CBT, focusing on how and why patients think and act in a given way in certain situations. In the program, patients are introduced to daily exercises and relevant information to help break the negative thoughts and the concept of the way we act. Patients receive weekly clinical asynchronous written support from their psychologist. A standard course of treatment lasts 10–12 weeks.

### 2.3. The implemented intervention

In 2018, CEDIP participated in the European Union's Horizon 2020 research project ImpleMentAll (IMA), where the ItFits-toolkit was developed, tested, and found to be effective (32). ItFits-toolkit was used in iPsych from December 2018 to May 2019.

The purpose of the ItFits-toolkit is to assist, design, and facilitate the implementation of eHealth interventions, with a special focus on iCBT. The toolkit provides a step-by-step framework for tailoring implementation strategies to the users' needs and capabilities. The toolkit is divided into four steps: (1) Identify and prioritize implementation goals and potential barriers to being able to achieve those goals. (2) Match the identified barriers with a range of evidence-based strategies for overcoming them. (3) Design and adapt the implementation strategies to meet the needs of the people and organizations you are working with. (4) Review and apply the implementation strategies and their impact in practice. This is a dynamic process in which teamwork is essential. Toolkit users work with an Implementation Core Team (ICT) and regularly consult with stakeholders to co-create the implementation strategy. The toolkit provides the users with ideas, methods, and guidance in doing so (24).

When using the ItFits-toolkit in iPsych, the primary problem area identified by the ICT was the screening of the group of self-referred patients for the clinical service; ensuring that the patients who seek treatment by the clinical service are eligible for it based on the inclusion and exclusion criteria. The main barrier identified by ICT was the request form. This form is completed by patients when they refer themselves to the service. To meet the goal of including an eligible patient group, the request form is an important point for optimization since in some cases it lets patients progress in the treatment system even though they are not eligible. The chosen strategy was to implement an automatic stopping point in the request form for the clinical service. Potentially, the strategy would be effective in reducing the inclusion of patients who do not match the service and redirecting them elsewhere, lessening the need for unnecessary assessments. This strategy was also selected because the automatic stopping point will let patients know early on that they should seek treatment elsewhere if they are not in the target group for this particular treatment offer. An automatic stopping point in the request form could, therefore, be expected to reduce time expenditure by patient and therapist alike. The intervention was implemented on 8 November 2019. A summary of the request form can be viewed in Table 1.

**Table 1:** Request form (summary).

Information about the patient	- Personal and contact information (name, address, civil status, kids, level of education, source of income). - How did the patient find the program? - How is the patient's access to digital necessities?
Treatment form	- Does the patient receive or wait for other treatment? - What disorder does the patient seek help for? - Is the patient searching for conversation- or text-based treatment? - Has the patient been diagnosed with and/or treated for a mental disorder? - Why did the patient search for this treatment? - What are the patient's expectations for the program?
Alcohol or drug abuse	- What is the patient's weekly alcohol intake? - Does the patient have a drug problem?
Current mental condition	- How long has the disorder affected the patient? - What has the frequency of problems been in the past 2 weeks? - Does the patient have a phobia? Which? - What is the severity of the phobia? - What is the patient's level of satisfaction with life, mental health, and physical health?
Social situations	- To what degree do different social situations influence the patient (5-point Likert scale with 20 situations)? - How many panic attacks has the patient experienced in the past week, and what was the severity of them? - How much has the patient worried about having a panic attack and/or avoided situations/activities that could lead to one in, the past week? - How much has the panic attack restricted the patient's daily and social life?
Approval from the patient	- Does the GP know about the patient's mental condition? Why or why not? - Will the patient grant approval for the GP to be informed about this?
Rejection of the application	- Different answers to the patient in the light of a lack of digital necessities; receiving or waiting for treatment; searching for conversation therapy; alcohol or drug abuse; and having disorders iPsych does not treat.

### 2.4. Participants

The interview participant sample was a convenience sample consisting of accessible therapists. We conducted individual, semi-structured interviews with seven therapists from the iPsych clinic, five female and two male therapists. At the time of the interview, they were aged between 27 and 41 years, with a mean age of 32 years. They had been working in the clinic for between 1 and 5 years, with a mean duration of 2 years. Their primary assignments were assessment and treatment of patients in the clinic. One therapist was licensed, and the others were supervised by a licensed psychologist. The therapists had all experienced both pre- and post-intervention.

The survey sample consisted of a subgroup of the IMA sample from the Danish trial site. Surveys were sent out to 37 on-site iPsych therapists, administrative staff, and off-site professionals who had contact with the target demographic and could inform patients about the iPsych treatment program. Surveys were completed by 10 of the 37 therapists, administrative staff, and off-site professionals. Three were men and seven were women. The participants were aged between 25 and 58 years old, with experience ranging from <1 year to more than 15 years.

### 2.5. Data collection

Qualitative data were collected through semi-structured, individual interviews with the therapists. The interviews were conducted using an interview guide with questions based on CFIR (33, 34). CFIR is a comprehensive meta-theoretical determinant framework, based on a review of 19 other theories, intended to be used to collect data from individuals who influence and impact implementation outcomes. The CFIR outlines five major domains each with multiple constructs that have the potential to influence implementation. The seven interviews lasted between 32 and 67 min with a median of 41 min, and were all audio-recorded and subsequently transcribed. The updated CFIR domains and constructs can be seen in Table 2 and the interview guide is shown in Appendix 1.

**Table 2:** CFIR domains and constructs (34).

**Domains**		**Constructs**
1	**Innovation** The “thing” being implemented, e.g., a new clinical treatment, educational program, or city service.	A. Innovation Source B. Innovation Evidence-Base C. Innovation Relative Advantage D. Innovation Adaptability E. Innovation Trialability F. Innovation Complexity G. Innovation Design H. Innovation Cost
2	**Outer setting** The setting in which the Inner Setting exists, e.g., hospital system, school district, state. There may be multiple Outer Settings and/or multiple levels within the Outer Setting (e.g., community, system, state).	A. Critical Incidents B. Local Attitudes C. Local Conditions D. Partnerships and Connections E. Policies and Laws F. Financing G. External Pressure 1. Societal Pressure 2. Market Pressure 3. Performance-Measurement Pressure
3	**Inner setting** The setting in which the innovation is implemented, e.g., hospital, school, city. There may be multiple Inner Settings and/or multiple levels within the Inner Setting, e.g., unit, classroom, or team.	A. Structural Characteristics 1. Physical Infrastructure 2. Information Technology Infrastructure 3. Work Infrastructure B. Relational Connections C. Communications D. Culture 1. Human Equality-Centeredness 2. Recipient-Centeredness 3. Deliverer-Centeredness 4. Learning-Centeredness E. Tension for Change F. Compatibility G. Relative Priority H. Incentive Systems I. Mission Alignment J. Available Resources 1. Funding 2. Space 3. Materials and Equipment K. Access to Knowledge & Information
4	**Individuals** The roles and characteristics of individuals.	**Roles subdomain:** A. High-level Leaders B. Mid-level Leaders C. Opinion Leaders D. Implementation Facilitators E. Implementation Leads F. Implementation Team Members G. Other Implementation Support H. Innovation Deliverers I. Innovation Recipients **Characteristics subdomain:** A. Need B. Capability C. Opportunity D. Motivation
5	**Implementation process** The activities and strategies used to implement the innovation.	A. Teaming B. Assessing Needs 1. Innovation Deliverers 2. Innovation Recipients C. Assessing Context D. Planning E. Tailoring Strategies F. Engaging 1. Innovation Deliverers 2. Innovation Recipients G. Doing H. Reflecting & Evaluating 1. Implementation 2. Innovation I. Adapting

Quantitative data were collected during the implementation of the ItFits-toolkit, which was based on a Stepped Wedge Cluster Randomized Controlled Trial (SWT) design (24). Specifically, survey data were collected in 10 waves at 3-month intervals during the study period using a specially designed functionality in the ItFits-toolkit (35). To measure the degree to which therapists perceived normalization through the intervention, data were collected using the Danish version of the Normalization Measure Development (NoMAD) questionnaire. The NoMAD questionnaire is designed to assess and evaluate the adoption, implementation, and integration of interventions into routine practice and is based on the NPT (25). The questionnaire is self-reported and has been developed and validated to assess NPT's constructs of coherence, cognitive participation, collective action, and reflexive monitoring. The 20 NoMAD items elicit assessments of implementation factors relating to the NPT constructs from individuals whose work is affected by the implementation. Items are scored on a 5-point Likert scale (1 = strongly agree, 5 = strongly disagree) (24). In addition, the Organizational Readiness for Implementing Change questionnaire (ORIC) was used to measure the degree of therapists' psychological and behavioral readiness for change (36). ORIC is a 12-item Likert scale instrument, where each of these 12 items is scored using a 5-point Likert scale (1 = “Strongly disagree”, 5 = “Strongly agree”). The questionnaire reflects organizational members' shared belief in their collective ability to implement a change, where a higher score indicates a higher willingness to execute one.

### 2.6. Data analysis

The transcribed interviews were analyzed using the General Inductive Approach (GIA) (37). GIA is often used in health science and was chosen as it offers a straightforward method of systematic analysis of qualitative data to have findings linked to emerging assessment questions. GIA is a generic method, where findings should be developed inductively from the themes in the raw data without restraints from structured methodologies. Investigator triangulation (38) was used, and three independent coders, authors SB, SLN, and KT, summarized the main themes of the transcripts based on the CFIR domains. Regarding constructs embedded in the content under these domains, data analysis was inductive and carried out through multiple readings of raw text, which was interpreted into a condensed summary format. The coders decided which data to use and which not to. Here, a text segment may have been coded in more than one sub-theme, and some may not have been coded at all if not relevant to the domains. Essential quotations were translated from Danish to English and added. The coders assessed reliability by checking each other's coding. If the coders disagreed, the matter was discussed until an agreement was reached. No software was used.

Quantitative data from the NoMAD and ORIC questionnaires were utilized to supplement the qualitative findings regarding the therapists' degree of normalization of the intervention and readiness to use it. The statistical analysis was conducted using data from respondents who had completed both the sixth- and tenth-wave questionnaires. The statistical tests were performed with STATA 17. For the tests related to normalization, the overall normalization score was used as the outcome variable, and time (in terms of waves) was used as the exposure variable. The sixth-wave questionnaire was used as the baseline, as this wave represented Implementation As Usual (IAU). The baseline was compared with the tenth wave, which was measured at the 6-month follow-up. Due to the small sample size, descriptive statistics (Ms, SDs, and score range) were used to assess the difference in the overall temporal effect of the normalization level. A low NoMAD score indicated that the therapists perceived a low level of normalization in the use of the new screening procedure, which could be caused by the low adoption of the intervention integrated into practice. Conversely, a high NoMAD score indicated that the therapists perceived a high level of normalization in the use of the new screening procedure. In addition, a Hedges' g-test was performed as a standardized measure of effect to describe the magnitude of the effect of time. The result of Hedges' g would be interpreted as 0.2 = small effect size, 0.5 = medium effect size, and 0.8 = large effect size. Descriptive statistical analysis (means, SDs) was also performed on the overall score from the ORIC data to examine the therapists' psychological and behavioral readiness to change at the baseline.

### 2.7. Ethics

The study was conducted in accordance with the ethical standards of the institutional and national research committees and with the 1964 Helsinki Declaration and its subsequent amendments or comparable ethical standards. Since the study was solely based on interview and questionnaire data collected from therapists, the Regional Committees on Health Research Ethics for Southern Denmark were informed about the study, but, in accordance with the Danish national ethical guidelines, no ethical approval was needed. All participants gave written informed consent. The study was reported to the Danish Data Protection Agency.

## 3. Results

The analysis of the transcribed interviews resulted in a description of the implementation process according to the CFIR domains. Each domain with the analyzed constructs and themes is presented in Table 3 and described in detail below, followed by the survey results.

**Table 3:** Analyzed domains, constructs, and themes.

**Domains**	**Constructs**	**Themes**
Innovation	Innovation design	
	Innovation relative advantage	More targeted patient group
		Supporting screening
		Supporting assessment interviews
		Better prepared patients
		Flipped clinic
	Innovation Trialability	
Outer setting	Local attitudes and conditions	
	External policies and incentives	
Inner setting	Structural characteristics, communications, and culture	
	Tension for change	
Individuals		
Implementation process		

### 3.1. Innovation domain

All therapists described several ways in which the screening intervention had influenced service delivery, mainly in terms of innovation design, relative advantage, and trialability. The most prominent aspects are discussed in more detail below.

#### 3.1.1. Innovation design

Four therapists preferred the design of the new request form compared to the old one. The therapists felt that, gradually, as more patients needed to be included in the treatment offer and more therapists were hired, a more structured screening process was needed, to ensure that patients received results based on equal screening procedures leading to optimal treatment offers. An important finding was that therapists felt that the structural changes that had been made had resulted in an increased sense of security about who to include and who to exclude, for example, because of suicide risk. They had found the system to be more intuitive, allowing them to get a feel for the individual patient more easily, resulting in increased visibility, collaboration, and a more evenly distributed caseload among therapists. Therapists found that the new request form, which automated the screening process, was well-designed and resulted in a shorter course for ineligible patients. In the old process, the patients would apply, have an assessment interview, wait for their treatment to be addressed at a clinical conference, and then receive a rejection. Now, patients fill out a more thorough treatment request form and based on the answers, ineligible patients receive a message to contact their GP for screening and evaluation. Since the new request form differentiates between anxiety and depression diagnoses, they wondered if it would be less of a burden to complete, especially for those patients suffering from depression, if they did not have to fill out the anxiety questionnaires in the tool. They felt that, compared to the old procedure, the instruments in the new request form had a more dynamic structure, causing a greater proportion of patients for whom the treatment was not relevant to be automatically referred back to their GP in the request form. One of the therapists spoke for all when they pointed out that: “*It was really easy and manageable, and you would quickly get insight into the patient's life and symptoms. Afterward, it was easier to have these facets elaborated on those areas where it seemed there were problems. So yes, definitely, it is much more pinpointed; it was designed for our patients, that is, the target group we offer treatment to. And it makes it a lot easier, and then I just think that, well, my experience has also been that those patients who made it to the assessment interview were more suited for our treatment course than earlier”* (Therapist 7). Accordingly, compared to the old system, the new request form enabled an easier, more valid, and more meaningful screening process, from the perspective of both patients and therapists, who also received better clinical data output. In addition, the new system created less administrative work because it was uploaded directly into the record, which helped reduce the waiting list.

#### 3.1.2. Innovation relative advantage

Compared to the old procedure, all therapists had experienced how the new request form was an improvement, in particular in terms of ensuring the uptake of a more targeted patient group, supporting screening and assessment interviews, and preparing patients for their treatment course. They believed that this resulted in a flipped clinic, releasing more time for more relevant treatment activities. The different aspects of the new request form's relative advantage will be covered in more detail in the following sub-sections.

##### 3.1.2.1. More targeted patient groups

Specifically, three therapists had experienced that the implementation of the new request form had resulted in improved targeting of the target population. The positive effect was seen in a more suitable group being screened eligible for assessment. It was important to the therapists that relevant patients are offered treatment; therefore, the target group description becomes crucial during the assessment interview, where the therapists have limited time to assess whether the patient should be offered one. It was the therapists' perception that the new request form made a difference during the assessment interviews, as they had spent less time evaluating the more severe groups of patients with more complicated symptoms. One therapist explained: “*Overall, I think that more relevant inquiries have come through. In general, it has been clear that the ones who came to the online assessment interviews were more likely to fit into the treatment offer. Then there was the need for a diagnostic interview to determine whether this was the right place for that patient. Before the new request form was put into use, we saw many more patients suffering from a personality disorder or something else that quickly excluded them from the treatment offer. So, I definitely felt that it became a more targeted group we got in through the sluice after the new request form was put into action”* (Therapist 1). As such, the new request form was seen as a help in optimizing intake and directing ineligible patients to more relevant treatment offers.

##### 3.1.2.2. Supporting screening

Two therapists found that the new request form had made it easier for them to screen new patients before offering them an assessment interview. They believed that the intervention had the desired effect of targeting a more eligible group of patients, thereby reducing the time spent on screening. They felt that the updated anxiety and depression questionnaires had streamlined the request form, making it easier to refer non-relevant patients to their GPs. The therapists felt that this had lightened the burden for the screening team when they assessed the relevance of the self-referrals to the iPsych offer, leading to fewer discussions about complex cases at clinical conferences and faster decision-making. Due to screenings based on the new request form, the therapists had experienced receiving a more relevant group of patients for assessment interviews from the screening team.

##### 3.1.2.3. Supporting assessment interviews

Six therapists experienced how the new request form supported their assessment of patients during the assessment interviews. The therapists felt that the optimization of intake procedures in the new request form resulted in patients invited to the assessment interview being more relevant to the treatment offer, which made the therapists more efficient during the assessment interviews. The therapists also explained that the new request form had a larger data output for them to use in preparing for assessment interviews and was better structured, which meant that they had to spend less time on preparation than before. Having more information available and knowing more about the patients' symptoms before the assessment interview made the therapists feel better prepared. For them, the request form had removed complexity in advance and allowed for a more thorough initial impression of the issues, for which the patient was seeking help to tackle. As one of the therapists explained: “*When we see the patient for the first time, we already have prior knowledge, and we have it from this request form. That means that you can already begin to target the session, for example: is this about anxiety? Is it about depression? Or both? And I know that already from the request form because the patient has to indicate that. In the request form, they get; they already fill that part out so I can begin to see where the problem is. What kind of anxiety is it? In what situations does it occur? Is it free-flowing, so it comes out of the blue, or is it triggered by specific situations or events? So, I already know that, and I actively use that prior knowledge in the session so that we can quickly start with the most relevant questions, where the suffering is. That is also what they need to work on in the course of their treatment”* (Therapist 4, shortened). For the therapists, this facilitated their assessment task and led them to feel better prepared to make decisions about the inclusion of the patients in the treatment offer.

##### 3.1.2.4. Better prepared patients

Three therapists felt that the new request form helped to prepare the patients for the treatment offer they were about to accept. It was significant to the therapists that the patients had already given consent and been informed about the inclusion and exclusion criteria in the request form. The therapists believed that this had given the patients who showed up for the assessment interviews a better sense of what they could expect from the treatment and prepared them for the structure and content of the treatment course. This again made the patients more able to decide beforehand whether the offer was a treatment course they could be motivated to take. This made it easier for the therapists to form a relationship with the patient, resulting in more assessment interviews with a positive atmosphere, more enthusiasm, and increased motivation. The therapists felt that they could use this relationship to motivate the patient, have a good process, and more easily maintain the patient–therapist alliance in an asynchronous iCBT treatment program. For the therapists, clarity and structure for both patient and therapist were important factors in the treatment delivery. It was the therapists' experiences that this had ensured a lower dropout rate from the service.

##### 3.1.2.5. Flipped clinic

All therapists agreed that an optimization of the digital request form had a positive effect on treatment by freeing up time for other relevant activities during the treatment. These activities were: management, administrative work, answering questions, giving feedback to the patients who are in a treatment course, and receiving feedback from their colleagues on how to improve patient communication. The therapists had experienced that the more structured and streamlined request process meant that they could direct more of their time and mental resources to where it mattered the most. This is their assessment of whether the patient is benefiting from the treatment and their focus on forming a caring relationship with the patient to motivate them through the treatment process. They felt that this allowed them to have more time with the patients, whom they could help with the treatment offer and for whom they could make more of a difference. The therapists felt that the implementation of the new request form had made their work and role as psychologists easier, as they had more time to meet the real person behind the patient on the screen and make proper assessments. For them, it was motivating to see more patients who could benefit from the treatment offer coming through the screening. They felt that their work could be more targeted and that they could ask the patient more relevant questions during the limited time of the assessment interview. This is what one of the therapists said when asked if the new request form had made it easier and how it played together with the time aspect: “*Yes, well, time is one thing, but I almost think that it is more important that it is relevant. Well, you know, in the meeting with the patient. That is what we can spend our time on, and that is what is the most relevant. And you can say that it has something to do with time. Because we do not have unlimited time”* (Therapist 4). This, they felt, enabled them to better target the assessment interview and collect more relevant information to create more meaning for the patient. In fact, they estimated that it could be even more if the request form is further adapted in the future.

#### 3.1.3. Innovation trialability

Four therapists had experienced an increase in the number of patients they assessed as suitable for inclusion in the treatment programs. One of them estimated that it was about two times as many, but it was difficult to say for sure because it varied from week to week. Another explained that in comparison with the previous procedure, where about 40% of the patients invited to the assessment interviews were offered a treatment course, now almost all patients were included. Yet another reported that it was 50%. This was because fewer patients were offered an assessment interview than those who requested treatment. Because the request form resulted in a more targeted group of patients being invited to the assessment interview, relatively more assessed patients were offered a treatment course. As far as they remembered, the therapists felt that the patients who came for the online assessment interview had diagnoses that were more relevant to the treatment offer. One therapist recalled that not many patients were screened out. The effect of the innovation will be tested in a future publication.

The therapists had the following ideas for future actions with the purpose to reach a more eligible patient group: (1) targeted public information and communication to clarify the treatment offer. However, here they had two caveats: first, the patients should not receive too much information, resulting in them self-diagnosing themselves; and second, the waiting list did not have to become too long; (2) general education of the public about what anxiety and depression are and when treatment seeking is advisable; (3) information on the iPsych website to prepare the patients for the treatment course by clarifying the format, structure, and content of the treatment offer; (4) directing non-eligible patients to ask their GP for another offer in the hope that they will not try to circumvent the request form; (5) better information to the GPs and other relevant professionals about the iPsych service, so as to be able to refer the patients correctly; (6) allowing the patient to screen themselves before submitting the request form; (7) allowing the treatment to be supplemented with, e.g., video or audio recordings of exposure situations, exposure in virtual reality, and exposure in real life; and (8) the flexibility to add telephone and/or video sessions to treatment courses.

### 3.2. Outer setting domain

All therapists experienced that the implementation process of the new request form had been influenced by events and circumstances that took place in the outer setting. In particular, aspects from the two constructs “Local attitudes and conditions” and “External policies and incentives”.

#### 3.2.1. Local attitudes and conditions

One prominent aspect that influenced the implementation process was the local attitudes and conditions, which five out of seven therapists experienced as a factor contributing to the implementation of the new screening process at iPsych. In particular, this was often related to the type of patients requesting treatment, as the clinic's open self-referral nature allows all patients to apply for treatment, whether or not their disorder is relevant to the clinic's treatment. The therapists felt that this created a loophole, as a significant share of their patients often suffered from mental illnesses with needs too complex for the iCBT offer. Still, with the therapists being passionate about treating their patients, they invested time and mental resources in video-based assessment, diagnosing and referring such complex patients to treatment elsewhere, “*...making it a very heavy task with a very steep learning curve…”* (Therapist 5). In addition, the therapists explained that the patient's expectations and knowledge of the treatment and the actual treatment offered were often misaligned. At times, this led to disappointed or angry patients who “*...thought they were going to have video consultations with us so that it was going to be weekly online conversations, which is not the concept at all”* (Therapist 7).

#### 3.2.2. External policies and incentives

Another aspect of the outer settings that influenced the implementation process, was external policies and incentives. This was experienced by five out of seven therapists. In 2018, there was a political decision to roll out iPsych nationally, at first on a temporary basis until 2020, pending an evaluation of the provision to decide whether it should be made permanent. The therapists felt that this had created an external pressure, as they were situated at the intersection of political agendas, external expectations from other regions about patient uptake from their specific region, and general expectations about patient uptake, waiting list, and time spent on screening and caseload numbers. The therapists explained how they had experienced a political agenda when it came to public relations (PR) and communicating about iPsych as an efficient treatment program where patients could receive help quickly. One pressure was the external expectations from other regions about the number of patients from their regions. One therapist explained that it was decided that each region was responsible for creating awareness about the service in their specific geographic area. The therapist felt that this had resulted in skewed patient eligibility and uptake between the regions, as there was naturally more awareness about the offer in Southern Denmark, as the service had already existed there or 5 years.

Another form of pressure seemed to be the goals set for the number of assessment interviews and treatment courses offered. One therapist elaborated: “*It is estimated that half of those who have been to the assessment interview start treatment because of the interplay between the external and internal factors and the pressure there is on how many assessment interviews we should have. But there are still limited resources for how many treatment sessions we can actually have at the same time. Also, we are not allowed to have a waiting list of more than 30 days”* (Therapist 1). As such, the therapists felt that the pressure needed to be balanced to keep the caseload manageable.

The therapists had also experienced pressure from outer settings due to political agendas regarding the number and percentage of admissions. This pressure influenced the implementation process as it became important to receive political support for iPsych to become a permanent nationwide service. The therapists explained that during the process, iPsych focused on recruiting eligible patients, treating them, and reducing the waiting list. In this process, they felt that efficiency had been used as a concept, as explained by one of the therapists: “*Well, the national rollout may be a part of the reason why it felt even more important to become more efficient in some way and reduce the waiting list and make sure that the energy is used properly”* (Therapist 3). Another used concept was streamlining, which another professional elaborated on: “*So, there was an upper pressure to make the offer more streamlined and targeted if it is to prove its relevance and if the regions are to continue paying for it”* (Therapist 5). The focus on the fact that iPsych had to streamline their service to move from a temporary to a permanent status had a great impact on the therapists' work as they internalized the pressure and took responsibility for making the national service permanent. The same therapist continued: “*I think that taking responsibility could help us remain in the process. We are leading the way now to make sure that this can actually continue to be there for those who need it. It is a motivational factor to make sure that it is followed through”* (Therapist 5). An important point to note here is that in this way, outer factors almost became inner factors for the therapists.

### 3.3. Inner setting domain

All therapists indicated that the implementation process had been influenced by events taking place in the inner setting. This was true in terms of both structural characteristics, communication, culture, and tension for change. The most prominent aspects are discussed in more detail below.

#### 3.3.1. Structural characteristics, communications, and culture

As we saw in the previous paragraph, the therapists explained that different pressures from the outer setting became factors influencing the inner setting. They felt pressured because they did not feel able to keep up with the increased demand for assessment interviews. Therefore, it became a concern to lighten the workload and save resources, as explained by one of the therapists: “*I think that external factors had had an influence in terms of streamlining or at least narrowing of the specialization of the target group we are trying to reach. I think there were some financial considerations in terms of our efficiency as a team”* (Therapist 6). In this streamlining, time became an important factor, the therapist continued: “*I mean, we need to see more patients in less time. A patient is not supposed to take up as much time as when seeing a regular psychologist. The treatment offer was made to reduce the waiting time in private practices but also so that patients could receive help relatively quickly when their problem appeared, instead of being on a waiting list for 3 or 4 months to see a psychologist. And this we have been able to feel in this process—that is more patients in, see them faster, and shorter time to the individual because we have a greater patient load”* (Therapist 6). Other evident factors that had contributed to the streamlining were changes in managers, therapists, and treatment programs; the therapist explained further: “*The new treatment programs have turned out to make it all more efficient by gathering all the diagnoses on one platform, and the chat function works better, and the different treatment modules are more user-friendly, simple, and easier to understand, and that has led to easier treatment and greater clarity. That is also something that happened internally”* (Therapist 6). As such, this was seen by the therapists as a starting point for streamlining the iPsych offer.

#### 3.3.2. Tension for change

Several therapists had experienced how the general level of receptivity to the new request form had contributed to the implementation process as the tension for change within the organization was high and the situation was perceived as intolerable and needing change. The therapists explained that as the service went from regional to national and the number of patients seeking treatment increased, the screening process became too cumbersome and time-consuming. One therapist pointed out that: “*The old system or the old way of requesting (treatment) and sending out (invitation or rejection) letters had become too extensive when we became a national service, it could have been a full-time job”* (Therapist 2). Another aspect of it was the handling of patient information, where the therapists also felt the need for more structure. They explained that it was inefficient and probably only worked because there were so few patients before the national rollout. Another therapist explained: *If we had continued with the same structure as there was when I started, the spreadsheet would have been unmanageable and some patient data would have been erased. And all the disappointed patients and all the psychologists who were left with the feeling that they could never get anyone involved. I mean, this is of course an exaggeration, but that wave was not good and there was really a need for more structure. This structure contributed to a positive evaluation and iPsych becoming a permanent service”* (Therapist 7). It was important for the therapists that iPsych was a treatment service and not a screening service. They saw it as a necessity for their future work that a larger proportion of ineligible patients who request treatment are automatically referred back to their GP for an assessment of alternative treatment options, so as to reduce the screening and assessment burden in iPsych.

### 3.4. Individuals domain

Four therapists felt that the individuals involved in the implementation of the innovation had influenced the implementation process in terms of both their characteristics and their roles. They felt that the implementers had worked “behind the scenes” with them knowing, without them noticing; however, they were aware that something went on as one of the therapists explained: “*Well, I think the group did a lot of work without me. I have not put a lot of thought into it, because I have primarily been doing my job. So, it was a process that you get a glimpse of once in a while. But I knew that it was taking up space… I mean for others”* (Therapist 3). Thus, the therapists acknowledged that the process had been time-consuming for some of their colleagues. In addition, they expressed confidence in the implementers to handle the task on behalf of the whole group, as another therapist elaborated: “*In general, I think that it is nice that I did not know more about it, because it is not my business, really. Well, I trusted that the ones who were doing the work did a good job in terms of developing the request form. So, it did not become a common responsibility for the therapists, there was like a working group appointed”* (Therapist 6). The working group consisted of senior therapists, which made sense to the interviewed individuals, and it was their impression that the working group had greatly influenced the development of the request form. The same therapist suggested that this influenced ownership of the request form: “*It must have a sense of ownership to those who were involved in it. Also, the fact that they were able to put their mark or stamp on it that the patients who fulfill those criteria we ask, these are the patients we want to treat”* (Therapist 6). As we can see, the therapists trusted that the more experienced therapists were the most competent to develop and adjust the request form to ensure relevant questions aimed at screening for the specific diagnoses treated by the clinic were included.

After iterations of developing and adjusting the clinical content of the request form, the therapists felt that the engineer had transformed the request form into a useful technical solution for patients to fill out online. It was the therapists' perception that the engineer had been busy and actively involved the working group in this phase, as exemplified here by one of them: “*Well, I felt that the engineer ended up playing a major role in iPsych with all this, and especially with the request form. He was good at coming to us and getting clinical input. I feel that we were properly involved”* (Therapist 3). Here, the therapists had felt included and that their advice had been listened to. In addition, they had experienced clear communication at the time of the implementation of the new request form, as another recalled: “*I had a pretty good feeling about why we were doing this and what the point was and what it was supposed to do for us”* (Therapist 7). Here, the therapists viewed the communication about the new request form to be fulfilling.

Overall, the therapists found the implementers to be complementary, ensuring that clinical input and technical expertise worked well together, as assessed by one of them: “*I think that you can only get there if you have a good collaboration between the different backgrounds”* (Therapist 7). The therapists saw this complementary team as beneficial to both the development, adjustment, and implementation of the request form.

### 3.5. Implementation process domain

The last of the five domains had to do with the implementation process itself. About half of the therapists had felt included, and about half wished they had been more involved. The most salient aspects have been examined below.

Four of the therapists had experienced the implementation process as being positive, continuous, and resource-demanding, involving many colleagues, meetings, and discussions. It was particularly important to them that the collaboration between the different professionals involved in the work was open and inclusive. This was not only important for the definition of the patient group but also for the rhythm structure and wording of the questions in the request form. They had experienced openness to their input on the clinical content of the request form. They felt that the inclusion of their experience-based knowledge had qualified the choice of data needed to assess which patients should be offered an online screening session. Here, the time represented an issue. Because the innovation had to do with changing the screening tool used to enroll patients in the clinic and was therefore of great importance to the therapists, they felt very invested in the process. For them, a swift completion was less important than the properness of the outcome of the process, as illustrated by one therapist: “*Well, I think that the therapists had a great interest in getting the new request form implemented and then we can test it and adjust it during the process. We on the front line really needed this tool but having these meetings and testing slowed down the process, which was a good thing. Because it made the request form even more targeted. By the time we actually implemented it in November, we had a better tool and were able to adjust from there. But I think it helped ensure that the questions we were asking were generating the data that we really needed, and I do not think that it would have been as sharp if we had not taken these actions and meetings about what we really needed to know”* (Therapist 5). For them, the quality of the new request form was most important; they wanted it to be better than the old one. They felt that it had become significantly better, but that they had not yet reached the end goal. They wanted to keep working on the request form and target the self-referring patient group even more.

Five therapists expressed the wish to feel more involved in the implementation process. One of them explained that they felt the process was somewhat closed and had an optimization goal. For two of them, the beginning, and the end of the process with the request form were clear. They felt that they had not been introduced to the innovation before the changes were implemented. In addition, the process was not complete as they continued to implement changes to the request form. Another two of them explained that they felt that there were different interests at stake in the implementation process, depending on the optimal way to design the innovation. It was their experience that the questions of the request form were selected to reflect a research need rather than a clinical need. This, they felt, led to the choice of evidence-based questionnaires in the tool over clinical interests, which had left them feeling kept out of the selection process and therefore having less ownership over the request form. One of them also requested more introduction to the therapist's interpretation of the questionnaires in the request form.

### 3.6. Survey results

**Figure 1:**
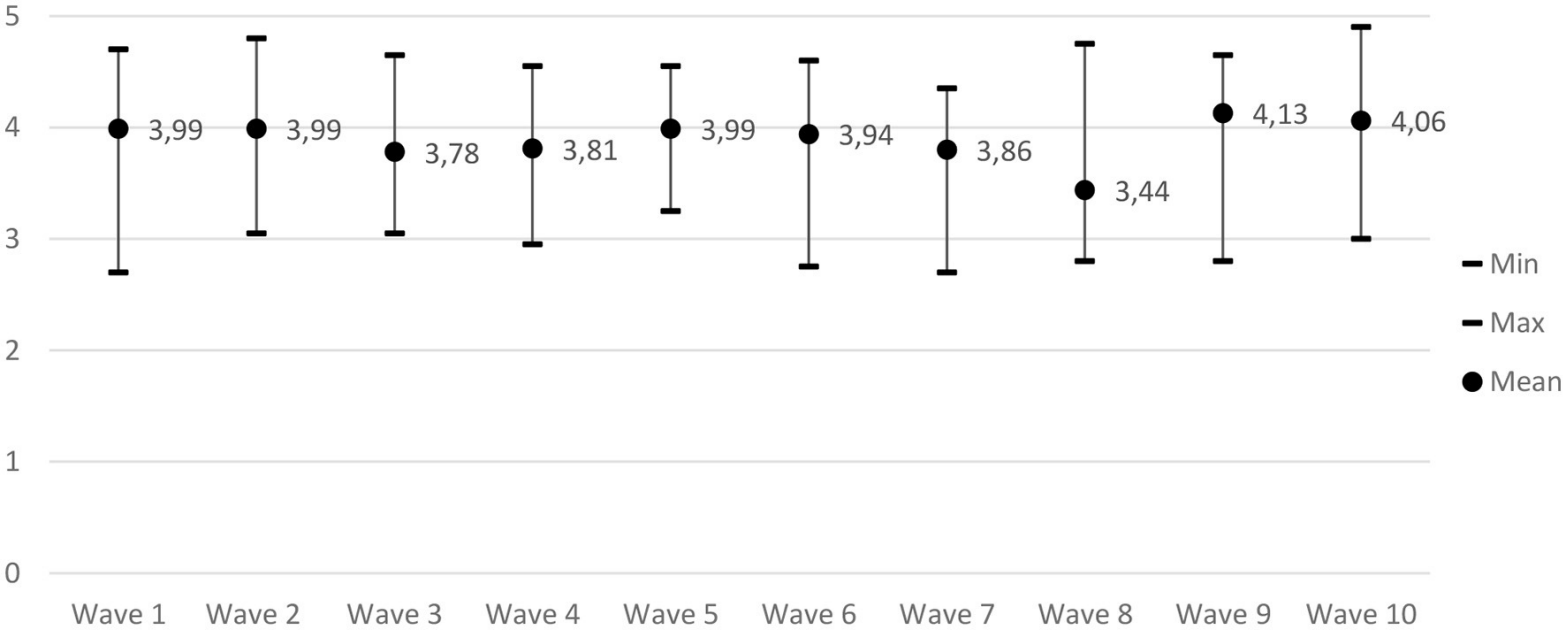
Overall NoMAD mean scores.

Table 4 shows the measures from the baseline compared with the follow-up measures. The overall mean score of NoMAD had a 0.12 positive change in mean from baseline to follow-up. Similarly, there was a positive change in both the minimum and maximum scores from baseline to follow-up. Furthermore, the Hedges' g-test showed that the effect size was g = 0.17, which is considered a small effect size according to the Hedges' g classification system. The g-effect sizes indicate that there was a higher level of normalization at the follow-up compared to the baseline with IAU. The normalization process is measured through waves, which are illustrated in Figure 1. As seen in Table 4 and Figure 1, there is a trend toward higher normalization during the waves. Moreover, the descriptive analysis of the ORIC survey showed the mean was equal to 4.5 with a score range of 2–4.58 at baseline. This indicates that the therapists assessed their organization's readiness index for the implementation of guided internet-based CBT for mild to moderate anxiety and depressive disorders at the high end at baseline. Overall, the results from the survey are reflected in the identified themes from the interviews.

**Table 4:** Baseline and 6-month follow-up NoMAD scores.

***Wave***	***N***	***M (SD)***	***Min*.**	***Max*.**
Baseline (sixth wave)	10	3.94 (0.60)	2.75	4.60
6-month follow-up	10	4.06 (0.67)	3.0	4.90

## 4. Discussion

The present study aimed to explore therapists' perceptions of the implementation of a new, more automated screening procedure using the NPT-based ItFits-toolkit in a specialized clinic offering guided iCBT for mild to moderate anxiety and depressive disorders. We collected and analyzed qualitative data using CFIR and quantitative data from NoMAD and ORIC questionnaires.

Regarding the CFIR innovation domain, we found that the implemented intervention had a facilitating effect on the implementation process. This finding is similar to the CFIR study by Hadjistavropoulos et al. (23), who also found that intervention characteristics [as they were called in the previous CFIR version (33)], together with implementation processes, had the greatest common positive impact on the implementation of iCBT. In their study, important factors that facilitated the iCBT implementation were: relative advantages, design quality, and strength of evidence of the iCBT program. In our study, the reason why the new request form was preferred over the old one was that it ensured the enrollment of a more targeted group of patients, supported screening and assessment interviews, and thus better prepared the patients for their treatment. This is similar to findings from a study on the implementation of online interventions by Kuso et al. (39), which found that screening in preventive interventions can be an important entry point for treatment. Furthermore, we found that therapists perceived that a greater proportion of assessment interviews had become patient courses. Taken together, these findings led to the emergence of a kind of flipped clinic, where the implementation of the new request form enabled time to be freed up for more relevant treatment activities. Other studies report flipped clinics, where the flipping is between online and face-to-face (23, 40). Here, the flipped element is more inspired by the flipped classroom theory, where using a digital element for easier tasks can free up more time for activities requiring the presence of teachers and students (41); in the present case, more time for more relevant treatment-related activities. Even more flipped digital elements are encouraged in future clinics to make the best use of available resources.

Regarding the CFIR outer setting domain, we found that the implementation process was facilitated by local attitudes and conditions. In comparison, a study by Hadjistavropoulos et al. (23), using CFIR to evaluate the implementation of iCBT in community mental health clinics found that the outer setting was less likely to facilitate implementation. In our study, the handling of patients played a particularly important role. Before the implementation of the intervention, therapists had experienced more patients who were disappointed with what was offered or had spent more time excluding patients who were too severely ill. This both caused and supported the implementation process, as it influenced the initial reasoning for the implemented intervention to streamline the screening process. The complex diagnostic nature of some patients helped the therapists to make sense of the implemented changes and their necessity, enhancing their sense-making, a critical element of normalization (25, 42, 43). In addition, events and circumstances that had to do with external policies and incentives influenced the implementation process. Here, we found that the therapists had experienced pressure from the political agenda and the goals set for uptake and inclusion in the clinic.

In relation to the CFIR inner setting domain, we found that the aforementioned pressure had created tension for change and facilitated the screening process to become more effective. Here, the implementation of a more structured intake procedure had been the clinic's answer to optimize and save resources. During this streamlining process, a feeling of ownership may explain the high degree of readiness to change (4.5), as indicated in the ORIC survey. Even though the ORIC survey showed a high mean value, the lower quartile was slightly low. In the interviews, some of the therapists implied that they did not feel they had an influence on the implementation process, which can explain why some indicated a low readiness to change. This was also seen in the study by Hadjistavropoulos et al. (23), where they, in contrast to this study, found the inner setting to be the greatest barrier to iCBT implementation. In their study, this was due to a lack of readiness for implementation because of limited resources and the perception that face-to-face constituted a better treatment offer.

Concerning the CFIR individual's domain, we found that the individuals involved in the implementation of the innovation had facilitated the implementation process in terms of both their characteristics and roles. In contrast, the study by Hadjistavropoulos et al. (23) had not found the individual characteristics to have clearly facilitated iCBT implementation. In our study, the implementers supplemented each other, ensuring that clinical input and technical expertise played well together. Working as a complementary team was of benefit for the development, adjustment, and implementation of the innovation. This finding is in line with a systematic review of barriers and facilitating factors when improving the implementation of eMental Health for mood disorders by Vis et al. (26), where the engagement of therapists in implementing and delivering an intervention was found to be related to the organizing structures, policies, and procedures within an organization. Furthermore, especially clear communication is highlighted as a facilitating aspect of our study. This finding is corroborated by a qualitative evaluation among therapists and managers on implementing guided iCBT for chronic pain and fatigue by Van der Vaart et al. (12). They found that internal communication was the most essential facilitator for effective implementation.

In terms of the CFIR implementation process domain, we found that the feeling of being included in an implementation process facilitated the process itself. Here, a positive, continuous, and resource-demanding process involving many colleagues, meetings, and dialogue, was a crucial factor. The overall NoMAD score in our study also indicated a high normalization of the use of guided iCBT for mild to moderate anxiety and depressive disorders at both baseline and 6-month follow-up. Compared with other studies that had used the NoMAD questionnaire to assess the degree of normalization, the respondents in our study indicated a high degree of normalization using guided iCBT (44–48). This can be explained by the respondents' high sense of being part of the implementation, which was also reflected in the interviews. This indicates that involving relevant users, such as the therapists, in the implementation process can create a higher degree of ownership and aid in the implementation of guided iCBT. This is in line with the CFIR study by Hadjistavropoulos et al. (23), where they also found that the engagement of different stakeholders in the implementation of iCBT facilitated the implementation process. In their study, implementation processes, together with intervention characteristics, had the greatest positive impact on the implementation of iCBT. Similarly, in another qualitative CFIR study on the identification of barriers and facilitators to the implementation of an Internet-based patient-provider communication service, Varsi et al. (27), together with the Van der Vaart study (12) also found that the therapists' attitudes were key and that their opinion of the implemented intervention affected the implementation process. The counterpart to this, i.e., not feeling involved in the process, was also experienced by some therapists in our study. Related to this finding, Van der Vaart et al. also reported that not all essential managers and other team members were involved in the implementation process. They found this lack of involvement of all stakeholders in the process to be a barrier to the implementation (12).

### 4.1. Strengths and limitations

The strength of this study is the use of mixed methods, which may provide more trustworthy findings (49). By using both interviews and surveys, we gained a more in-depth insight into how the implementation occurred in practice.

Even though the toolkit and the surveys were NPT based, it may be a strength that we chose the CFIR framework for qualitative data collection and analysis. These approaches have been shown to work well together in recent research, where the CFIR domains offer a comprehensive range of issues to explore, and the NPT contributes explanatory power (50). The relatively small sample size of seven interviews in the qualitative part may also be a limitation; however, Crouch and McKenzie (51) argue in a trend report that a small number of participants can be used for inductive analyses to facilitate the interviewer–interviewee connection and to increase the validity of a semi-structured interview. In addition, Guest et al. (52) learned in an experiment with data saturation and variability that the first six interviews were crucial for the emergence of basic elements for meta-themes. Therefore, they recommended six interviews as an adequate number to develop meaningful themes and useful interpretations. It is a strength that the data were coded by three independent coders, which increased internal validity and reliability (53) and thus the credibility of the analysis (37, 54).

Our results showing a higher degree of normalization, compared to other studies, may point to the fact that different studies use NoMAD data in different ways, making it difficult to compare results with other studies. The survey had a very low number of participants due to staff changes during the study, as some participants left the site and, therefore, no longer participated in the trial. Some of the participants also no longer wanted to participate in the trial due to a perceived lack of relevance in their answers. The small number of participants, which can be seen as a weakness, could have led to bias, as a small sample size may not be representative of the population, and there is a risk that the outcome is due to chance (55). This is somewhat compensated for by the 10 waves of data collection, which strengthened the sample. However, this has been taken into account by only using Hedges' g and descriptive analyses. Despite the small number of participants, the results of the surveys were reflected in the results of the interviews. This indicates that the quantitative results are valid, despite the small sample size. Furthermore, this also supports the validity of the experiences described in the interviews.

In conclusion, we found that the therapist's perceptions of using the ItFits-toolkit to implement a new and more automated screening procedure in the clinic pointed to the fact that the toolkit use added nuance to the process. The systematic and tailored elements in the toolkit influenced the ICT to find a solution customized to local conditions. This is in contrast to an expert-driven process, where an implementation intervention could have easily been automatically chosen, which would not have met local needs by default. Future studies should continue to explore implementation processes to ensure the most effective use of the clinical digital interventions developed, providing professional help to the growing population in need of mental health services.

## Data availability statement

The raw data supporting the conclusions of this article will be made available by the authors, without undue reservation.

## Ethics statement

Ethical review and approval was not required for the study on human participants in accordance with the local legislation and institutional requirements. The patients/participants provided their written informed consent to participate in this study.

## Author contributions

CV and JP-J designed and developed the ItFits-toolkit in the IMA Consortium. KT and KM designed the present local trial study. KT conducted the semi-structured interviews. SB, SN, and KT conducted the qualitative analysis. CD, TH, EJ, and KM conducted the statistical analysis. All authors contributed to writing the manuscript.
