# Supplementary material for: Therapist perceptions of the implementation of a new screening procedure using the ItFits-toolkit in an iCBT routine care clinic: A mixed-methods study using the consolidated framework for implementation research
Source: Front Psychiatry. 2023 Apr 6;14:1104301. doi: 10.3389/fpsyt.2023.1104301 (PMC10117952; doi:10.3389/fpsyt.2023.1104301)
Supplement: Supplementary file 1 [file Data_Sheet_1.docx]

| **Appendix 1. Interview guide** | |
| --- | --- |
| **Introductory questions:** | *Thank you for agreeing to participate in this interview. If you consent, I would like to record the interview. The interview will be about iPsychs process towards uptake of a more targeted patient group. I will pose some introductory questions, asking about you and your work role. Then I will pose the main questions, asking from five different perspectives about your experience of the process towards uptake of a more targeted patient group. I will finish off with a couple of concluding questions. I will start asking questions, you are welcome to explain and describe in the direction experiences and perceptions occur to you. First, the introductory questions.*   - May I ask about your sex and age? - When were you employed in iPsych? If stopped, when? - What are/were your tasks? - Are you an authorized psychologist? If yes, when were you authorized? - Are there anything else or something more you would like to tell me about your background? |
| **Questions related to iPsychs** **process towards the uptake of a more targeted patient group:** | *Then I will move on to the main questions. They ask from five different perspectives about your experience with how different factors have influenced the process towards uptake of a more targeted patient group, and in connection to that how the request form has been taken in use and has been used.*   - How have you experienced that the new request form have influenced the process towards uptake of a more targeted patient group (e.g., how is it to use, are there any advantages/disadvantages, how does the uptake work, time spent on screenings, patient trajectory)? - How have you experienced that the process towards uptake of a more targeted patient group has been influenced by factors outside the clinic (e.g., patient needs and the national roll-out of the clinic, are they interconnected)? - How have you experienced that inner factors in the clinic have influenced the process towards uptake of a more targeted patient group (e.g., the clinic’s situation the past years, the way you are organized, or the way you interact and communicate, are they interconnected)? - How have you experienced that colleagues involved in working with the new request form have influenced the process towards uptake of a more targeted patient group (e.g., personal characteristics, knowledge about/resistance against the change)? - How have you experienced that the process towards uptake of a more targeted patient group have been influenced by the activities and actions performed to put the new request form into use (e.g., the process and execution of the commissioning of the new request form, where, when, how, and by whom is the request form developed)? - Do you have anything else you would like to say regarding the process towards uptake of a more targeted patient group and the role of the request form connected to that? |
| **Concluding questions:** | *Then I will move on to the concluding questions.*   - Do you have any ideas for other actions that might support the process towards the uptake of a more targeted patient group? - Do you have anything else you would like to say? - Do you have any questions you would like to ask?   *Thank you very much for participating in this interview and sharing your experiences. The interview will be anonymized and transcribed. Then we will analyze data and publish the findings.* |
